# Correction: *Reduced BDNF expression in the auditory cortex contributed to neonatal pain-induced hearing impairment and dendritic pruning deficiency in mice*

**DOI:** 10.1136/rapm-2022-103621corr1

**Published:** 2026-02-25

Li N, Chen B, Jia G, *et al*. Reduced BDNF expression in the auditory cortex contributed to neonatal pain-induced hearing impairment and dendritic pruning deficiency in mice. *Reg Anesth Pain Med* 2023;48:85-92. doi:10.1136/rapm-2022-103621

In the originally published article, figure 4 contained unclear labeling that could lead to misinterpretation. The higher-magnification images in the upper portion of Figure 4D were intended as representative examples of BDNF co-localization with GFAP, NeuN, and IBA1 in the CFA+NS group only and were NOT used for quantitative analysis. The bar graph in the lower portion of figure 4D represents BDNF expression levels measured as mean integrated optical density (IOD), rather than co-localization.

Figure 4 has been corrected to clearly indicate the corresponding regions in panelsA–C, add appropriate group labels in Panel D, revise the Y-axis label, and specify scale bar sizes.

These corrections do not affect the results or conclusions of the study. The authors apologize for any confusion caused.

